# Correlation Between Hallux Valgus Severity and the Prevalence of Metatarsus Adductus in Hallux Valgus

**DOI:** 10.1002/jfa2.70049

**Published:** 2025-04-19

**Authors:** Yuan‐Shao Chen, Che‐Han Liang, Han‐Ting Shih, Kao‐Chang Tu, Shih‐Chieh Tang, Shun‐Ping Wang

**Affiliations:** ^1^ Department of Orthopaedic Surgery Taichung Veterans General Hospital Taichung Taiwan; ^2^ Department of Orthopaedics Tungs' Taichung MetroHarbor Hospital Taichung Taiwan; ^3^ Department of Materials Science and Engineering Feng Chia University Taichung Taiwan; ^4^ Department of Industrial Engineering and Enterprise Information Tunghai University Taichung Taiwan; ^5^ Department of Post‐Baccalaureate Medicine College of Medicine, National Chung Hsing University Taichung Taiwan

**Keywords:** hallux valgus, HVA, MAA, metatarsus adductus, prevalence

## Abstract

**Introduction:**

Hallux valgus (HV) is a common foot deformity, with metatarsus adductus (MA) identified as a potential predisposing factor. MA has been shown to negatively affect surgical outcomes for HV, particularly in severe cases. This study aims to clarify the prevalence of MA in the HV population using different metatarsus adductus angle (MAA) measurement methods and assess whether MAAs are influenced by HV severity.

**Materials and Methods:**

This retrospective study included 294 feet from 147 participants. Patients were classified into non‐HV (normal) and HV subgroups, with HV severity graded as mild, moderate, or severe based on the hallux valgus angle (HVA) measured on dorsoplantar weight‐bearing radiographs. The prevalence of MA was assessed using four radiographic measurements: Sgarlato's MAA (MAA4), modified Sgarlato's MAA (MAA5), modified Engel's angle, and the calcaneo‐second metatarsal angle (rearfoot‐MT2). The interclass correlation coefficient was used to evaluate the reliability of the measurements. The correlation between HVA and MAA was analyzed using Spearman's Rho coefficient, and the prevalence of MA was compared using various measures.

**Results:**

After excluding 87 feet, 207 feet (146 HV and 61 non‐HV) from 147 participants were analyzed. All four MAA measurements showed excellent reliability, with the modified Engel's angle demonstrating the highest interobserver reliability and strongest correlation with HVA. HVA was significantly higher in the MA (+) group compared to the MA (−) group (32.21 vs. 24.78° and *p* = 0.001). The overall prevalence of MA in the cohort was 19.3% (MAA4), 24.2% (MAA5), 18.4% (modified Engel's angle), and 8.2% (rearfoot‐MT2). MA was significantly more prevalent in the HV group compared to the normal group (24.0% vs. 4.9%) when using the modified Engel's angle, with MA prevalence increasing as HV severity worsened.

**Conclusions:**

MA is common among patients with HV, with its prevalence increasing in parallel with HV severity. The modified Engel's angle is a reliable and sensitive method for detecting MA associated with HV, particularly in severe cases, and its use can help tailor surgical plans to improve outcomes. Surgeons should be mindful of the presence of concomitant MA when planning HV surgery, as it may negatively affect surgical outcomes and increase the risk of recurrence.

## 1 Introduction

Hallux valgus (HV) is one of the most prevalent chronic foot conditions seen by foot and ankle specialists [1]. The overall prevalence of HV was estimated to be 23% in adults aged 18–65 years and 35.7% in elderly individuals over 65 years [2]. The severity of HV can be graded based on the degree of increase in the hallux valgus angle (HVA) and the first intermetatarsal angle (IMA) [3]. Although the exact biomechanical cause of HV remains unclear, several predisposing factors have been identified, including metatarsus adductus (MA), old age, female gender, a positive family history, and constricting footwear [4, 5, 6, 7].

MA is characterized by an oblique midtarsal joint and the medial deviation of the metatarsals relative to the midfoot in the transverse plane [8, 9]. MA have been suggested to predispose individuals to the development of HV deformity [10, 11, 12, 13, 14]. Patients with MA are 3.5 times more likely to develop HV [11], likely due to the loss of dynamic medial support provided by the abductor hallucis [15]. Additionally, MA can compromise the surgical correction of HV by limiting the available space for transposition of the first metatarsal head due to the varus position of the second metatarsal [16]. Unrecognized MA deformity has been reported to negatively impact the surgical outcomes of symptomatic HV, resulting in an increased revision rate and a greater likelihood of recurrence [4, 17, 18, 19, 20]. To obtain better outcomes, particularly in severe cases, addressing the association between MA and HV severity is crucial before performing surgical interventions for symptomatic HV.

Various methods have been suggested for detecting MA, including visual assessment (heel bisector method), radiographs, ultrasound, photocopier imaging, footprints, dynamic foot pressure analysis, and gait analysis [21]. Among these, radiographic evaluation is the most widely used method for diagnosing MA. Numerous radiographic assessments have been developed to measure the metatarsus adductus angle (MAA), including Sgarlato's MAA (MAA4), modified Sgarlato's MAA (MAA5), Engel's angle, modified Engel's angle, the talo‐first metatarsal angle, the calcaneo‐second metatarsal angle (rearfoot‐MT2), the calcaneo‐fifth metatarsal angle, Lepow's angle, and Kilmartin's angle [21, 22, 23]. Of these, four—MAA4, MAA5, modified Engel's angle, and rearfoot‐MT2—are noted for their relatively higher interobserver reliability, making them the recommended measurements for the accurate diagnosis of MA [22, 24].

MA is a common clinical condition, with a reported prevalence of 25%–55% among HV patients [4, 10, 18, 25, 26, 27]. However, there is considerable variation in the reported prevalence of MA within HV populations, which may be attributed to differing measurement techniques of the MAA [22]. Despite these observations, the true prevalence of MA in the HV population remains unclear. Additionally, the relationship between MAA and HV is still a matter of debate. Lee et al. found a significant correlation between MAA and HVA [28], whereas Dessouky et al. observed a positive trend between MAA and HVA, although it did not reach statistical significance [24]. Furthermore, the association between MAA and HV severity has been examined using the Manchester Scale, a tool with uncertain reliability, particularly in older HV patients. However, the relationship between MAA and HV severity, as defined by the HVA, has not been clearly established [29].

The purpose of this study is to clarify the prevalence of MA among the HV population using various measurement methods and to determine whether MAAs are influenced by the severity of HV. We hypothesize that different radiographic measurement techniques affect the prevalence results in both HV and non‐HV populations and that there is a positive association between MAA and HV severity. The findings of this study will provide clinicians with deeper insights into MA in the HV population and may help improve surgical outcomes for symptomatic HV, particularly in patients with severe MA.

## 2 Materials and Methods

### 2.1 Patient Recruitment

This study was approved by the Institutional Review Board of our institution. We retrospectively reviewed all medical records and images of patients who presented to our foot and ankle outpatient clinics between August 2013 and December 2022. The inclusion criteria were as follows: (1) age over 18 years, (2) HV patients who had undergone surgical treatment on either foot, (3) availability of weight‐bearing dorsoplantar (DP) and lateral view radiographs, and (4) radiographs of non‐HV feet from patients with other conditions. A total of 147 individuals (294 feet) met the inclusion criteria for further analysis. The exclusion criteria were as follows: (1) lack of weight‐bearing foot radiographs, (2) significant foot arthritis or deformity, (3) history of prior trauma, (4) infection, and (5) previous foot or ankle surgery.

### 2.2 The Grading of Severity of HV and Subgroups

The groups were classified based on the severity of HV according to the HVA measured on weight‐bearing DP radiographs. HV was diagnosed when the HVA exceeded 15°, and the cases were further categorized into four subgroups based on the severity of HV: normal (< 15°), mild (15°–30°), moderate (30°–40°), and severe (> 40°) [13].

### 2.3 Radiographic Measurements

**FIGURE 1:**
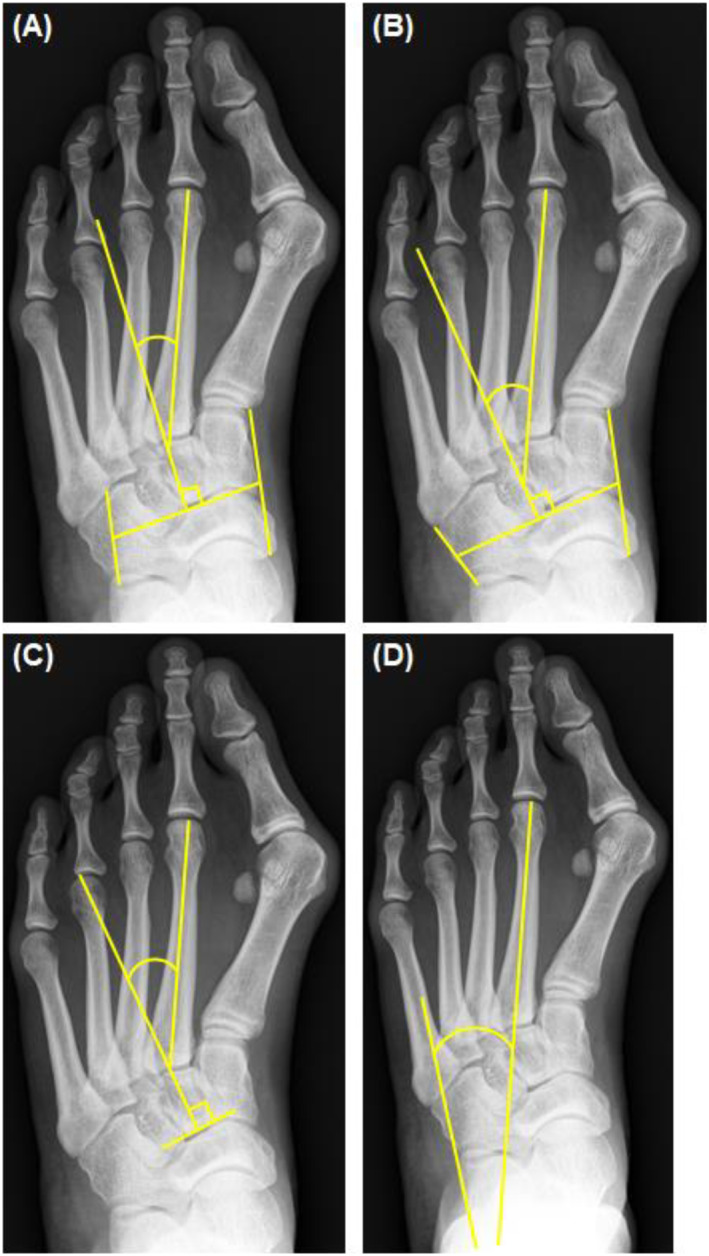
Illustration of the four radiographic measurements of metatarsus adductus angle (MAA). (A) Sgarlato's MAA (MAA4). (B) modified Sgarlato's MAA (MAA5). (C) modified Engel's angle. (D) calcaneo‐second metatarsal angle (rearfoot‐MT2).

All radiographs were taken at a single facility following a standardized radiographic protocol. The images were retrieved using the built‐in software of the picture archiving and communications system (PACS) with ultraquery technology (Taiwan Electronic Data Processing, Sindian City, Taiwan). Measurements were obtained from weight‐bearing DP and lateral‐view radiographs of the foot by two independent observers. The radiological parameters assessed included HVA, IMA, and four different measurements of the MAA, namely MAA4, MAA5, modified Engel's angle, and rearfoot‐MT2. The definitions of these radiographic measurements are as follows: HVA: the angle between the longitudinal axis of the first metatarsal and the first proximal phalanx [30]. IMA: the angle between the longitudinal axis of the first metatarsal and the longitudinal axis of the second metatarsal [30]. MAA4: the angle between the longitudinal axis of the second metatarsal and the longitudinal axis of the lesser tarsus using the fourth metatarso‐cuboid joint as a reference [22]. MAA5: the angle between the longitudinal axis of the second metatarsal and the longitudinal axis of the lesser tarsus using the fifth metatarso‐cuboid joint as a reference [22]. Modified Engel's angle: the angle between the longitudinal axis of the second metatarsal and a line perpendicular to the proximal articular surface of the middle cuneiform [22]. Rearfoot‐MT2: the angle between the longitudinal axis of the second metatarsal and the rearfoot reference line, which is a line parallel to the lateral border of the calcaneum [22] [Figure 1]. The diagnostic cutoff values for MA were derived from previously reported literature: MAA4 at 14°, MAA5 at 20°, modified Engel's angle at 24°, and rearfoot‐MT2 at 18° [22].

### 2.4 The Interclass Correlation Coefficient of MAA Measurements

To assess the interclass correlation coefficient (ICC) of the four MAA measurements, radiographs from 40 randomly selected patients were analyzed, with the patients evenly distributed into four subgroups representing different severities of HV (10 cases per subgroup). Two authors, both blinded to the outcomes and not involved in the operative team—one an orthopedic surgeon and the other a foot and ankle specialist—independently measured the target parameters on the radiographs to verify the reliability and reproducibility of the measurements. To evaluate intraobserver reliability, the foot and ankle specialist measured the same four angles again on the same 40 radiographs after a 2‐week interval. The ICC levels were categorized as follows: 0.01–0.20 (slight), 0.21–0.40 (fair), 0.41–0.60 (moderate), 0.61–0.80 (substantial), and 0.81–1.00 (excellent) [31].

### 2.5 Statistical Analysis

All variables were tested for the normality of data distribution using the Kolmogorov–Smirnov test. Continuous variables are presented as medians (interquartile ranges, IQRs), whereas categorical variables are expressed as frequencies (percentages). Differences between groups were evaluated using the Mann–Whitney *U* test for continuous data and the chi‐squared test for categorical variables. Correlations between subgroups were assessed using Spearman's Rho coefficient, and differences across two or more independent sample subgroups were analyzed with the Kruskal–Wallis test, followed by a post hoc Dunn–Bonferroni test. All statistical analyses were conducted using SPSS version 22.0 (IBM, New York, NY, USA), with a *p* value of < 0.05 considered significant.

## 3 Results

### 3.1 Patient Demographics

After excluding 87 feet based on these criteria, a total of 207 feet from 147 individuals were included in the final analysis. Among the excluded cases, 47 had prior foot or ankle surgeries, 16 had arthritis or rheumatoid arthritis, 14 had a history of trauma, 8 lacked weight‐bearing foot radiographs, and 2 had Müller–Weiss syndrome. The final analysis included a total of 207 feet from 147 individuals, comprising 61 non‐HV feet and 146 feet with mild to severe HV. There was no significant difference in the proportion of the left and right feet across the groups. All groups demonstrated a predominance of female participants, with no significant differences between the groups in regard of sex. However, age showed significant differences across the groups [Table 1]. Post hoc analysis revealed that patients in the moderate and severe groups were significantly older compared to those in the mild group (*p* < 0.05).

**TABLE 1:** Patient demographics and characteristics.

	Normal	Mild	Moderate	Severe	*p* value
Patient (*N*)	46	32	35	34	
Age	57.5 (51.8–65.0)	45.5 (35.0–56.8)	59.0 (35.0–69.0)	64.0 (53.3–69.5)	< 0.001**
Sex					0.328
Female	32 (69.6%)	27 (84.4%)	25 (71.4%)	28 (82.4%)	
Male	14 (30.4%)	5 (15.6%)	10 (28.6%)	6 (17.6%)	
Feet (*N*)	61	56	49	41	
Side					0.976
Right	27 (44.3%)	26 (46.4%)	23 (46.9%)	20 (48.8%)	
Left	34 (55.7%)	30 (53.6%)	26 (53.1%)	21 (51.2%)	

*Note:* Chi‐squared test or Kruskal–Wallis test.

** *p* < 0.01.

### 3.2 ICC of MAA Measurements

The intraobserver and interobserver reliabilities of the four MAA measurements are presented in Table 2. The ICCs for all four measurements were above 0.9, indicating excellent reliability for both intraobserver and interobserver assessments.

**TABLE 2:** Intraobserver and interobserver reliabilities of the MAA measurements.

	Intraobserver				Interobserver			
	ICC	95% CI		*p* value	ICC	95% CI		*p* value
		Lower	Upper			Lower	Upper	
MAA4	0.989	0.979	0.994	< 0.001**	0.992	0.980	0.997	< 0.001**
MAA5	0.960	0.926	0.979	< 0.001**	0.989	0.980	0.994	< 0.001**
Modified Engel	0.977	0.956	0.988	< 0.001**	0.989	0.977	0.995	< 0.001**
Rearfoot‐MT2	0.988	0.978	0.994	< 0.001**	0.985	0.971	0.992	< 0.001**

Abbreviations: CI, confidence interval; ICC, interclass correlation coefficient; MAA, metatarsus adductus angle; MAA4, Sgarlato's MAA; MAA5, modified Sgarlato's MAA; Rearfoot‐MT2, calcaneo‐second metatarsal angle.

** *p* < 0.01.

### 3.3 The Difference in HVA and IMA Between Patients With and Without MA

To assess the presence of MA and its correlation with the HVA and IMA, participants were categorized into MA (−) and MA (+) groups based on specific cutoff values suggested using the previous literature: 14° for MAA4, 20° for MAA5, 24° for the modified Engel's angle, and 18° for rearfoot‐MT2. Among the 207 feet in the cohort, MA (+) was identified in 40 feet (19.3%) using MAA4, 50 feet (24.2%) using MAA5, 38 feet (18.4%) using the modified Engel's angle, and 17 feet (8.2%) using rearfoot‐MT2. Notably, the HVA are higher in MA (+) groups compared to MA (−) groups across all four measurements subgroups. However, significant differences were only observed in groups measured using MAA4, the modified Engel's angle, and rearfoot‐MT2 but not MAA5. In contrast, no significant differences in IMA were detected across any of the classification criteria when comparing MA (−) and MA (+) groups [Table 3].

**TABLE 3:** Comparisons of HVA and IMA between MA (−) and MA (+) groups.

	*N* (%)	HVA	*p* value	IMA	*p* value
MAA4			0.021*		0.783
MA (−)	167 (80.7%)	25.49 (12.72–35.71)		12.15 (9.29–16.00)	
MA (+)	40 (19.3%)	32.15 (18.96–42.51)		12.18 (9.81–16.17)	
MAA5			0.100		0.886
MA (−)	157 (75.8%)	25.55 (12.87–36.88)		12.15 (9.27–16.00)	
MA (+)	50 (24.2%)	31.67 (17.76–41.50)		12.18 (9.71–16.00)	
Modified Engel			0.001**		0.213
MA (−)	169 (81.6%)	24.78 (12.49–36.51)		12.00 (9.25–16.00)	
MA (+)	38 (18.4%)	32.21 (26.54–43.15)		12.89 (11.32–15.87)	
Rearfoot‐2MT			0.003**		0.293
MA (−)	190 (91.8%)	25.52 (12.95–36.72)		12.00 (9.37–16.00)	
MA (+)	17 (8.2%)	34.84 (31.50–45.33)		13.18 (12.13–15.49)	

*Note:* Mann–Whitney *U* test.

Abbreviations: HVA, hallux valgus angle; IMA, the first intermetatarsal angle; MA, metatarsus adductus; MAA4, Sgarlato's MAA; MAA5, modified Sgarlato's MAA; Rearfoot‐MT2, calcaneo‐second metatarsal angle.

* *p* < 0.05.

** *p* < 0.01.

### 3.4 Comparisons of MAA Values in Different Severities of HV

**FIGURE 2:**
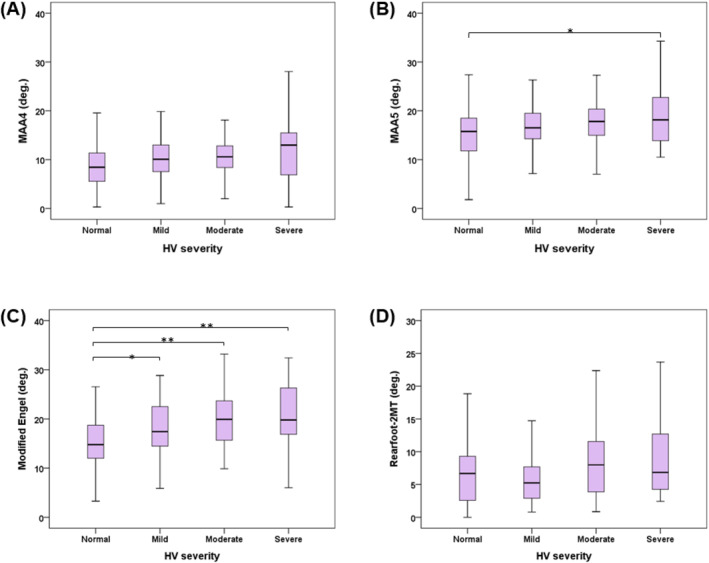
Comparisons of metatarsus adductus angles (MAAs) between different severities of hallux valgus (HV). (*p < 0.05 and **p < 0.01).

All four measurements of the MAA varied significantly across different severities of HV as determined using the Kruskal–Wallis test (*p* < 0.05). An increasing trend in MAA values was observed corresponding to the severity of HV across all four measurement subgroups as shown in Table 4. Further post hoc analysis using the Dunn–Bonferroni method revealed a significant difference (*p* < 0.05) between the normal group and the mild, moderate, and severe groups when applying the modified Engel's angle. However, when using MAA5, a significant difference was observed only between the normal and severe groups as depicted in Figure 2.

**TABLE 4:** Differences in MAAs across varying severities of HV.

	Normal	Mild	Moderate	Severe	*p* value
MAA4	8.45 (5.02–11.59)	10.08 (7.54–13.13)	10.57 (8.39–12.83)	12.97 (6.29–15.54)	0.026*
MAA5	15.77 (11.78–18.73)	16.51 (14.23–19.52)	17.82 (14.91–20.41)	18.16 (13.72–22.91)	0.028*
Modified Engel	14.76 (11.62–18.75)	17.43 (14.33–22.55)	19.92 (15.67–23.90)	19.79 (16.75–26.33)	< 0.001**
Rearfoot‐2MT	6.69 (2.53–9.69)	5.25 (2.88–7.83)	7.99 (3.53–11.62)	6.85 (4.06–12.91)	0.024*

*Note:* Kruskal–Wallis test.

Abbreviations: HV, hallux valgus; MAA, metatarsus adductus angle; MAA4, Sgarlato's MAA; MAA5, modified Sgarlato's MAA; Rearfoot‐MT2, calcaneo‐second metatarsal angle.

* *p* < 0.05.

** *p* < 0.01.

### 3.5 Correlations Between HVA, IMA, and MAAs

Among the four measurement methods, the MAAs showed significant correlations with the HVA in all subgroups, whereas no significant correlation with the IMA was observed in most subgroups. The strongest correlation between MAAs and HVA was observed in the modified Engel's measurement subgroup [Table 5].

**TABLE 5:** Correlation between MAAs with HVA and IMA.

	HVA		IMA	
	*r*_*s*_	*p* value	*r*_*s*_	*p* value
MAA4	0.203	0.003**	0.011	0.878
MAA5	0.197	0.004**	0.029	0.675
Modified Engel	0.315	< 0.001**	0.207	< 0.001**
Rearfoot‐2MT	0.157	0.024*	−0.004	0.951

*Note:* Spearman's Rho coefficient.

Abbreviations: HVA, hallux valgus angle; IMA, the first intermetatarsal angle; MAA, metatarsus adductus angle; MAA4, Sgarlato's MAA; MAA5, modified Sgarlato's MAA; Rearfoot‐MT2, calcaneo‐second metatarsal angle.

* *p* < 0.05.

** *p* < 0.01.

### 3.6 Comparisons of MA Prevalence in Different Severities of HV

**FIGURE 3:**
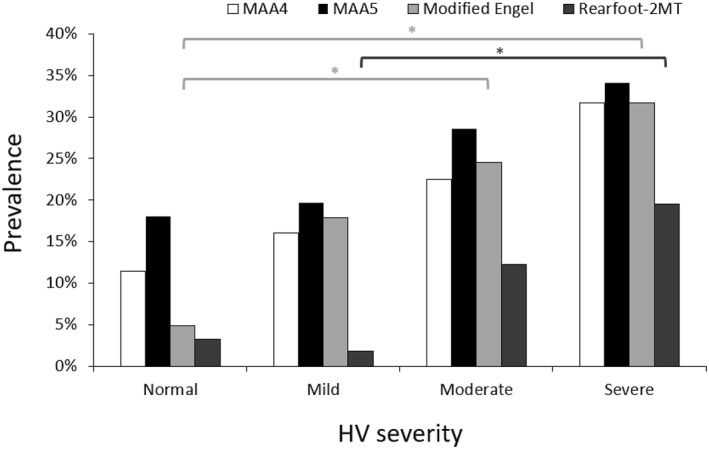
The prevalence of metatarsus adductus (MA) across different severities of hallux valgus (HV). (*p < 0.05).

The overall prevalence of MA was higher in the HV group compared to the normal group across all MAA measurements. Notably, a significant difference between the two groups was detected only when using the modified Engel's angle (*p* = 0.001). A clear increasing trend in MA prevalence was observed as HV severity progressed across all four measurement methods, but significant differences were found specifically in the modified Engel's angle and rearfoot‐2MT subgroups. Specifically, MA prevalence measured using the modified Engel's angle in the normal, mild, moderate, and severe HV groups was 4.9%, 17.9%, 24.5%, and 31.7%, respectively, with statistically significant differences (*p* = 0.004). However, no significant differences were observed using MAA4 and MAA5 as shown in Table 6. Additionally, post hoc analysis confirmed that the differences in MA prevalence between the normal group and both the moderate and severe groups, as measured using the modified Engel's angle, were statistically significant (*p* < 0.05). In contrast, a significant difference was observed only between the mild and severe groups when measured using rearfoot‐MT2. In comparison to normal participants, the prevalence of MA is significantly higher in the severe HV group across all four measurement techniques. Furthermore, no significant differences were detected between the normal and mild groups across any of the four measurement techniques as illustrated in Figure 3.

**TABLE 6:** Prevalence of MA across normal and HV groups with varying severities of HV.

Feet (*N*)	Normal	HV	*p* value
	61	146	
MAA4	7 (11.5%)	33 (22.6%)	0.065
MAA5	11 (18.0%)	39 (26.7%)	0.183
Modified Engel	3 (4.9%)	35 (24.0%)	0.001**
Rearfoot‐2MT	2 (3.3%)	15 (10.3%)	0.095

Feet (*N*)	Normal	Mild	Moderate	Severe	*p* value
	61	56	49	41	
MAA4	7 (11.5%)	9 (16.1%)	11 (22.4%)	13 (31.7%)	0.068
MAA5	11 (18.0%)	11 (19.6%)	14 (28.6%)	14 (34.1%)	0.201
Modified Engel	3 (4.9%)	10 (17.9%)	12 (24.5%)	13 (31.7%)	0.004**
Rearfoot‐2MT	2 (3.3%)	1 (1.8%)	6 (12.2%)	8 (19.5%)	0.005**

*Note:* Chi‐squared test.

Abbreviations: HVA, hallux valgus angle; IMA, the first intermetatarsal angle; MAA, metatarsus adductus angle; MAA4, Sgarlato's MAA; MAA5, modified Sgarlato's MAA; Rearfoot‐MT2, calcaneo‐second metatarsal angle.

** *p* < 0.01.

## 4 Discussion

This study aims to clarify the prevalence of MA in the HV population using various measurement methods and to assess whether MAAs are influenced by HV severity. A total of 207 feet (146 HV and 61 non‐HV) from 147 participants were analyzed, with a predominance of female participants and a significant correlation between HV severity and age. The four MAA measurement methods (MAA4, MAA5, modified Engel's angle, and rearfoot‐MT2) demonstrated excellent reliability. HVA were significantly higher in MA (+) groups compared to MA (−) groups, and MAAs showed significant correlations with HVA, with the strongest correlation observed between modified Engel's angle and HVA. The prevalence of MA was higher in the HV group compared to the normal group, indicating that MA prevalence is influenced using the measurement technique and correlates with HV severity. These findings provide deeper insights into the relationship between HV and MA.

In this study, higher HVA values were consistently observed in MA (+) groups across all four measurement methods, and the prevalence of MA increased with the severity of HV. The overall prevalence of MA in the cohort was 19.3%, 24.2%, 18.4%, and 8.2%, whereas among the HV group, the prevalence was 22.6%, 26.7%, 24.0%, and 10.3% when measured using MAA4, MAA5, modified Engel's angle, and rearfoot‐MT2, respectively. Previous research has reported the prevalence of MA in HV populations to be 55% when measured using Engel's angle [10], 33% measured using MAA4 [32], and 29.6% measured using MAA5 [25]. Our findings are consistent with the data measured using MAA4 and MAA5 but differ from the prevalence evaluated using Engel's angle. Furthermore, Dawoodi et al. reported significant variability in the prevalence of MA in HV patients when measured using different techniques, with rates of 70%, 62%, 25.3%, 25.3%, and 45% observed for MAA4, MAA5, modified Engel's angle, rearfoot‐MT2, and Engel's angle, respectively [22]. The prevalence of MA measured using MAA4 and MAA5 was notably higher in their study compared to our findings. Additionally, hindfoot deformity may influence MAA when measured using rearfoot‐MT2, potentially explaining the variance between their reported prevalence (25.3%) and ours (8.2%). Our data align more closely with the reported prevalence of MA measured using the modified Engel's angle.

In HV cases with a large MAA, instability of the 1st TMT joint should be considered [26], as higher surgical recurrence rates of up to 29.6% have been reported [18]. Multiple surgical approaches, including 1st TMT arthrodesis [19] or abduction osteotomy of the lesser toes, may be necessary in severe MA cases [27, 32]. Early detection of MA before surgery is crucial for improving outcomes. Among the various techniques for measuring MAA, MAA4 and the modified Engel's angle have the highest ICCs and rearfoot‐MT2 has the lowest interobserver reliability [22], with the modified Engel's angle showing superior reliability compared to MAA4 [24]. The strongest correlation between MAA and HV severity was observed with the modified Engel's angle, with a prevalence of MA exceeding 31.7% in the severe HV group. Additionally, this method detected significantly higher MA prevalence in moderate and severe HV cases compared to the normal group. The modified Engel's angle offers high reliability, ease of measurement, and greater sensitivity in detecting MA changes in severe HV cases.

Regarding the measurement of MAA, multiple methods have been proposed, all of which have demonstrated satisfactory ICC performance [8, 21]. Most of the literature commonly references MAA4, MAA5, Engel's angle, and modified Engel's angle [33]. Dawoodi et al. suggested that the five techniques—MAA4, MAA5, Engel's angle, modified Engel's angle, and rearfoot‐MT2—are reliable, with most showing an ICC between 0.85 and 0.92 [22]. The original Engel's angle is defined as the angle between the longitudinal axis of the second metatarsal and a line bisecting the middle cuneiform [23], and it was modified by Thomas et al. in 2006 [34]. In this study, the modified Engel's angle was preferred over the traditional Engel's angle due to its simplicity, ease of use, and reliable performance. When comparing the Engel's and modified Engel's angles, the latter demonstrates higher intraobserver ICC (0.92 vs. 0.90) and interobserver ICC (0.91 vs. 0.84) [22]. In the current study, all four measurements (MAA4, MAA5, modified Engel's angle, and rearfoot‐MT2) showed ICC values above 0.9, indicating excellent reliability. These results are comparable to those reported by Dawoodi et al., which were 0.93, 0.87, 0.91, and 0.87, respectively [23].

Our findings regarding the influence of age and gender on the presence of HV in this cohort are consistent with existing literature. A meta‐analysis of 45 studies reported that HV is more prevalent in females and individuals over 60 years of age, with pooled prevalence rates of 23.74% for females and 11.43% for males. The prevalence also increases with age, with 11% in those younger than 20 years, 12.22% in adults aged 20–60 years, and 22.7% in individuals over 60 years [35]. Similarly, Roddy et al. [7] found that HV is associated with age (adjusted OR 1.61 per decade and 95% CI 1.52–1.69) and female sex (adjusted OR 2.64 and 95% CI 2.26–3.08). The age‐related increase in HV is often attributed to changes in joint mechanics and plantar loading patterns, which reduce loading under the hallux as the deformity progresses, thus impacting the propulsive function of the first metatarsophalangeal joint [36]. Furthermore, the risk of surgical recurrence is significantly higher in older patients, with those aged 60 years or older having a fivefold increased risk compared to younger patients under 50 years [37]. Our data align with these findings, as patients in the severe and moderate groups were significantly older than those in the mild group, with median ages of 64.0, 59.0, and 45.5 years, respectively.

The severity of HV in this cohort was assessed using the HVA rather than the IMA, as existing evidence suggests that IMA may be underestimated when the second metatarsal is adducted toward the first metatarsal in the presence of MA [6, 23]. To correct for this inaccuracy caused by metatarsal abduction, the “true IMA” should be adjusted by adding Engel's angle to the IMA and then subtracting 15 degrees [38]. Moreover, the correlation between the MAA and HVA has been shown to be stronger than that between MAA and IMA. As a result, the authors chose HVA as the criterion for defining HV severity. Additionally, Kilmartin et al. demonstrated a significant correlation between HVA and MAA (*r* = 0.26 and *p* < 0.01), whereas no significant correlation was found between IMA and MAA (*r* = −0.2 and *p* > 0.05) [39]. Similarly, Banks et al. reported a linear correlation between increasing juvenile HVA and increasing MAA [40]. The findings of this study are consistent with these previous reports.

This study has several limitations. First, it was conducted in a single institute, which may limit the generalizability of the results to other populations. Second, the retrospective design of the study inherently presents challenges, such as potential selection bias and reliance on the accuracy of medical records and radiographs. When surgeons apply these results to clinical settings, it is essential to fully consider the possible selection bias potentially introduced using the retrospective study design as well as the external validity due to the data being from a single‐center study. Third, the cohort was limited to patients who presented to a foot and ankle outpatient clinic, potentially excluding less symptomatic or undiagnosed individuals with HV. To mitigate these limitations, future multicenter and prospective studies with a more diverse patient cohort are recommended to validate and further expand our findings.

## 5 Conclusions

MA is common among HV patients, with its prevalence increasing alongside HV severity. HV and MA are closely related and can negatively impact each other's prognosis. Surgeons preparing for HV surgery should consider the presence of concomitant MA. The modified Engel's angle is a reliable and clinically valuable technique for assessing MA, particularly in severe HV cases, as it demonstrates excellent intraobserver and interobserver reliabilities, a strong correlation with HVA, and significant associations with MA prevalence. Early detection of MA using this method can aid in surgical planning, improve outcomes, and reduce postoperative recurrence, especially in patients with pronounced structural abnormalities.

## Author Contributions

All listed authors contributed substantially to this work. All authors have read and agreed to the published version of the manuscript. **Yuan‐Shao Chen:** conceptualization, methodology, data curation, visualization, writing – original draft, writing – review and editing. **Che‐Han Liang:** investigation, formal analysis, writing – original draft, writing – review and editing. **Han‐Ting Shih:** software, writing – review and editing. **Kao‐Chang Tu:** project administration, writing – review and editing. **Shih‐Chieh Tang:** supervision, writing – review and editing. **Shun‐Ping Wang:** conceptualization, methodology, resources, visualization, writing – original draft, writing – review and editing.

## Ethics Statement

This study was conducted in accordance with the Declaration of Helsinki and approved by the Institutional Review Board of our institute (No. CE14084). Patient consent was waived due to the retrospective design of image study and the regulations of the Institutional Review Board.

## Conflicts of Interest

The authors declare no conflicts of interest.

## Data Availability

The data that support the findings of this study are available from the corresponding author upon reasonable request.
